# Indwelling Catheters Should Be Restricted in Primary and Revision Arthroplasty: A Retrospective Analysis After Changes to Hospital Standard Perioperative Treatment Protocol

**DOI:** 10.3390/antibiotics14040368

**Published:** 2025-04-02

**Authors:** Matthias Schnetz, Tim Jakobi, Larissa Ewald, Alexander Klug, Matthias Münzberg, Yves Gramlich

**Affiliations:** 1 Department of Trauma and Orthopaedic Surgery, BG Unfallklinik Frankfurt am Main, 60389 Frankfurt, Germany; matthias.schnetz@bgu-frankfurt.de (M.S.); tim.jakobi@bgu-frankfurt.de (T.J.);; 2 Department of Orthopaedics and Trauma Surgery, Agaplesion Markus Hospital, 60431 Frankfurt, Germany

**Keywords:** arthroplasty, revision arthroplasty, indwelling catheter, urinary tract infection, urinary retention, periprosthetic joint infection

## Abstract

**Background:** Indwelling catheters are used in the perioperative management of patients treated with total joint arthroplasty (TJA) to facilitate fluid control, ease postoperative miction until patients are able to ambulate, and prevent postoperative urinary retention (POUR). However, in TJA, they may be associated with a higher risk of urinary tract infections (UTIs). The aim of the study was to analyze the rates of urinary tract infections and POUR. **Methods:** Between 2021 and 2022, this study retrospectively identified patients before and after a change in the hospital standard perioperative treatment protocol towards a more restrictive use of indwelling catheters for TJA. In 2021, the use of indwelling catheters involved standard care, but the use was restricted in 2022. **Results:** A total of 1521 patients were included: 636 patients (41.8%) underwent primary arthroplasty, 646 (42.5%) underwent revision arthroplasty, and 239 (15.7%) underwent hip arthroplasty for femoral neck fractures. Standard use of indwelling catheters significantly decreased from 62.0% to 38.0% (*p* < 0.001), and the rate of UTI was significantly lower after the protocol change (4.7% vs. 1.2%; *p* < 0.001). Perioperative urine catheterization was a significant risk factor for UTI (OR = 4.22; *p* < 0.001), and UTI was a significant risk factor for PJI (OR = 9.99; *p* < 0.001). POUR increased slightly from 0.9% to 1.8%, but the difference was not significant. POUR was mostly diagnosed following the exchange of the acetabular component in revision arthroplasty (n = 11; 52.4%). **Conclusions:** Indwelling catheter use was associated with high rates of UTIs. Restricting perioperative use of indwelling catheters was effective in preventing UTIs while causing only a moderate increase in easily treatable postoperative urinary retention. Therefore, the use of indwelling catheters should be avoided in arthroplasty whenever possible.

## 1. Introduction

Indwelling catheters were routinely used in patients undergoing total joint arthroplasty in the early days to facilitate fluid control and ease postoperative miction until patients were able to ambulate. However, controversies persist in the literature regarding indwelling catheter use due to risks of postoperative urinary tract infections (UTIs) and surgical site and periprosthetic joint infections [1]. Advances in total joint arthroplasty have resulted in reduced surgery times, decreased blood loss, and fast-track protocols promoting early ambulation. In hip joint arthroplasty for femoral neck fractures, time to surgery is associated with increased complications and mortality, resulting in faster surgical treatment and, therefore, faster time to ambulation [2,3]. Several systematic reviews and meta-analyses of randomized controlled trials have reported that indwelling catheter use in primary arthroplasty is associated with a higher rate of UTI, leading to recommendations against routine catheterization in total joint arthroplasty [4,5]. As a result, stricter indications for indwelling catheterization in primary and revision arthroplasty were introduced in our study center.

Patients undergoing surgery without indwelling catheters are at risk of postoperative urinary retention (POUR). The risk depends on various factors, including older age, male sex, spinal or epidural anesthesia, and surgical time [6,7,8]. Reported rates of postoperative urinary retention vary between 0 and 50% [6,9,10]. To avoid complications, routine or limited scanning protocols for urinary retention have been discussed [11].

In revision arthroplasty, surgical time and blood loss can vary and, in some cases, be more difficult to predict. However, there is limited evidence on the use of indwelling catheters in revision arthroplasty and the rates of UTI and postoperative urinary retention.

**This study aimed** to determine the rates of UTI and urinary retention in primary and revision arthroplasty before and after changing the perioperative treatment protocol from standard use to restricted use of indwelling catheters. Furthermore, cases of UTI or urinary retention were analyzed.

## 2. Results

A total of 1521 patients were included in the study. Two groups were retrospectively analyzed: in 2021, the standard care involved routine use of indwelling catheters in perioperative treatment, whereas in 2022, their perioperative use was restrictive. In the first year, 466 (62.0%) received an indwelling catheter compared to 292 (38.0%) in the second year, reflecting a significant decrease (χ^2^(1) = 87.56; *p* < 0.001). Patients’ demographic and surgical data are included in Table 1.

**Figure 1:**
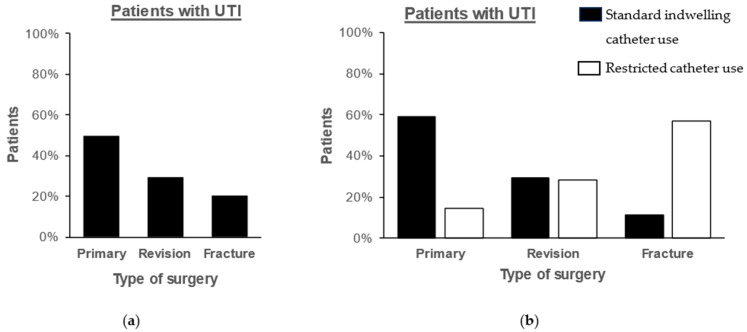
Type of surgery of patients with urinary tract infection (UTI; n = 34) in (**a**) the entire study period and (**b**) in the year before and after changes in hospital guidelines.

UTIs were diagnosed in 42 (2.8%) patients. Eight of these patients (0.5%) were treated for periprosthetic joint infection and were excluded from the analysis. There was a significant decrease in the rate of UTI from 4.7% (27 of 574 patients) before to 1.2% (7 of 608 patients) after the hospital guidelines restricted the use of urinary catheterization (χ^2^(1) = 13.27; *p* < 0.001). Perioperative urinary catheterization was significantly associated with an increased risk of UTI using a univariate logistic regression model (odds ratio [OR] = 4.22; 95% confidence interval [CI] 1.83–9.77; *p* < 0.001). The rate of UTI significantly reduced in primary procedures following the restricted use of indwelling catheters (χ^2^(1) = 14.50; *p* < 0.001; Figure 1). Furthermore, a multivariable logistic regression model was used to determine risk factors for UTI (χ^2^ = 31.077; *p* < 0.001; Table 2). Variables with significant influence on the risk of UTI included urinary catheterization (OR = 3.45; 95% CI 1.53–7.74; *p* = 0.003) and arthroplasty of the hip (OR = 9.09; 95% CI 1.43–1.67; *p* = 0.011). Microbiological data of patients with UTI are included in Table 3, and resistance patterns are included in Supplementary Table S1. After developing UTI, three patients (8.8%) were diagnosed with a periprosthetic joint infection with bacterial specimens of tissue samples matching bacteria of UTI in two of the three cases (*Enterococcus faecalis*, *Proteus mirabilis*). UTI was a significant risk factor for PJI (OR = 9.99; 95% CI 2.65–37.59; *p* < 0.001).

**Figure 2:**
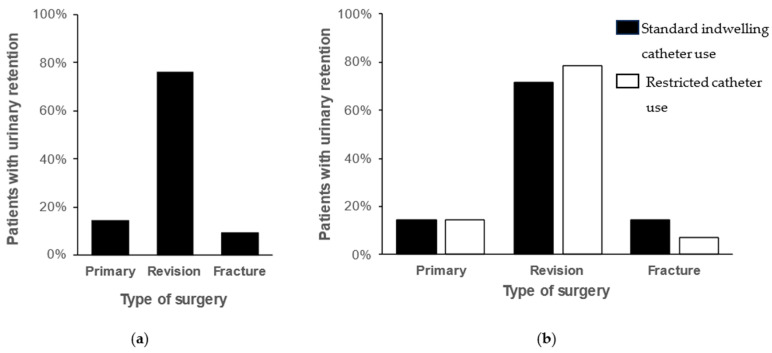
Type of surgery of patients with urinary retention in (**a**) the entire study period and (**b**) in the year before and after changes in hospital guidelines.

During the study period, 21 (1.4%) patients were diagnosed with urinary retention. The number of patients with urinary retention increased from 7 (0.9%) before restricting the use of indwelling catheters to 14 (1.8%) after restriction, but the difference was not statistically significant (χ^2^(1) = 2.21; *p* = 0.137). The use of indwelling catheters was significantly associated with a reduced postoperative urinary retention rate using a univariate logistic regression model (0.1% vs. 2.6%; OR = 0.05; 95% CI 0.01–0.37; *p* = 0.003). A multivariable logistic regression model was used to determine risk factors for urinary retention (χ^2^ = 67.39; *p* < 0.001; Table 4). Variables with significant influence on the risk of urinary retention included urinary catheterization (OR = 0.02; 95% CI 0.003–0.17; *p* < 0.001), arthroplasty of the hip (OR = 2.17; 95% CI 2.82–166.67; *p* = 0.003) and type of surgery with an increased risk after revision arthroplasty compared to primary arthroplasty (OR = 16.02; 95% CI 4.47–57.44; *p* < 0.001). Most patients diagnosed with urinary retention had revision surgery (Figure 2).

Among patients diagnosed with urinary retention, the three most common procedures were exchange of the acetabular component (n = 11; 52.4%), primary hip arthroplasty (n = 3; 14.3%), and exchange of mobile components or revision for periprosthetic joint infection (each n = 2; 9.5%). Only one patient (4.8%) diagnosed with urinary retention previously underwent knee arthroplasty (Table 5). All patients were successfully treated with in/out or Foley catheters, followed by successful catheter removal. Male gender was not a risk factor for urinary retention (see Table 4). Additionally, no patient with postoperative urinary retention had previous events of urinary retention or pre-existing urological conditions. However, all patients with urinary retention underwent general anesthesia.

Using a multiple linear regression model (adjusted R^2^ = 0.003; *p* = 0.038), neither UTI (OR = 1.83; 95% CI −0.88–4.54; *p* = 0.186) nor urinary retention (OR = 4.30; 95% CI = 0.50–8.11; *p* = 0.067) had a significant influence on the length of hospital stay

## 3. Discussion

In this study, a change in hospital standard perioperative protocol towards a more restrictive use of indwelling catheters significantly reduced the use of perioperative catheterization as well as postoperative UTI.

UTI after total joint arthroplasty of the hip or knee is a significant risk factor for surgical site infections and periprosthetic joint infections [1,12]. While some publications fail to find a correlation between UTI, especially preoperative UTI, and periprosthetic joint infections, other publications have identified UTI as an independent risk factor [12,13]. This discrepancy may be explained by the definition of UTI, as preoperative asymptomatic bacteriuria does not increase the risk of periprosthetic joint infection [13,14]. However, preoperative symptomatic UTIs within 1–2 weeks prior to total joint arthroplasty and postoperative UTIs significantly increase the risk of periprosthetic joint infection [1,13,14,15]. Therefore, any UTI should be treated before primary and revision total joint arthroplasty whenever possible [14]. In this study, no patient in the primary and revision arthroplasty group underwent surgical treatment with preoperative UTI. Notably, because indwelling catheter use increases the risk of UTI in total joint arthroplasty, catheterization should be avoided whenever possible to prevent surgical site and periprosthetic joint infections [5]. In this study, restricting the use of indwelling catheters in total joint arthroplasty significantly decreased UTI rates from 4.7% to 1.2%, especially in primary arthroplasty. The number of patients with UTI who underwent primary arthroplasty decreased (60% vs. 14%); however, a concomitant rise in patients who underwent fracture arthroplasty was observed (11% vs. 57%), which may be explained by the lower decrease in catheterization rate in this group. Furthermore, UTI was a statistically significant risk factor for periprosthetic joint infection in this study, which has only limited significance due to the small number of cases (n = 3), but corroborates with previous findings [1]. Several studies have analyzed the relationship between pathogens causing UTI and surgical site infections, whereby all studies were only able to show a partial match of pathogens [16,17]. In this study, pathogens were identical in two of the three patients with postoperative UTI and PJI; however, the pathogens might be of different strains. Nevertheless, restricting the use of indwelling catheters is an effective strategy to prevent postoperative urinary tract and, based on published literature, subsequent periprosthetic joint infections. However, side effects such as urinary retention must be considered.

Postoperative urinary retention may occur after surgical treatment without catheterization. The potential risk factors of postoperative urinary retention include administration of nonsteroidal anti-inflammatory drugs [6], spinal and epidural anesthesia [18], male gender, benign prostatic hypertrophy, and duration of surgery [7,19]. However, in this study, male gender was not a risk factor for postoperative urinary retention. The literature on risk factors for postoperative urinary retention shows conflicting results, often identifying several risk factors in univariate analysis that are not confirmed in the multivariate analysis, suggesting a multifactorial etiology [6,20]. The type of anesthesia is one of the most discussed risk factors of urinary retention in total joint arthroplasty. Epidural and spinal anesthesia are discussed as risk factors for postoperative urinary retention [5,7], while general anesthesia has not been found to have any influence on the rate of urinary retention [19]. However, a randomized controlled trial by Miller et al. found no significant difference between patients undergoing total hip arthroplasty with spinal anesthesia with or without indwelling catheters [21]. In this study, most patients had general anesthesia, and none underwent epidural anesthesia. Therefore, data on the type of anesthesia were not available. However, no significant influence can be expected in this study, given that all patients with postoperative urinary retention underwent general anesthesia. Similar to the results of a meta-analysis of randomized controlled trials, there was a slight increase in postoperative urinary retention after a change in perioperative treatment protocols, but the difference was not statistically significant [5]. When analyzing the surgical procedure of patients with urinary retention, 76.2% underwent revision arthroplasty, and 52.4% of the patients had a revision of the acetabular cup. This might be explained by longer operative times and intraoperative intravenous infusions in revision arthroplasty compared to primary arthroplasty. However, given the number of revision procedures included in this study, a urinary retention rate of 1.4% is low compared to previously published rates of 0.1–50.0%, with most studies reporting rates for primary arthroplasty [5]. Therefore, operative times and intravenous infusions alone do not explain the postoperative urinary retention rate, as a higher rate would be expected given the number of revision procedures. This highlights the multifactorial nature of postoperative urinary retention, which is not yet fully understood [20]. To our knowledge, there is no literature on the risk of postoperative urinary retention by type of revision arthroplasty. One explanation might be that all patients who underwent acetabular cup revision were limited to partial weight bearing, whereas revision of the stem often facilitates full weight bearing. However, Shapiro et al. did not identify postoperative weight bearing as a risk factor for urinary retention [22].

While surgical site and periprosthetic joint infections pose a major medical and economic burden [23], the outcome of postoperative urinary retention is scarcely discussed in the literature. Postoperative urinary retention can lead to cardiovascular symptoms, bladder overdistension, and urinary tract infection [24], highlighting the need for prompt detection and treatment of postoperative urinary retention. In a recent study, Magnuson et al. demonstrated the superiority of a symptom-based screening compared to routine screening of all patients [11], a method also applied in this study. All patients included in the study were successfully treated with in/out catheterization or Foley catheters, which were removed before hospital discharge. In primary arthroplasty, some studies show an increase in length of stay due to postoperative urinary retention [7]. In this study, neither UTI nor postoperative urinary retention affected the length of hospital stay. This might be an effect of the general higher length of stay in revision arthroplasty, making up 42.5% of all cases included in this study.

This study has some limitations. Patients were retrospectively included, which has some inherent limitations. Patient comorbidities were not available in the database and could, therefore, not be included in the study. Therefore, a comprehensive risk factor analysis was not possible. However, patients with postoperative urinary retention were followed up for comorbidities, none of whom presented with potential risk factors for urinary retention. Furthermore, in the second group, after the change in perioperative standard protocol on the use of indwelling catheters, catheterization was limited to certain cases when deemed necessary by the surgeon or anesthesiologist, which may lead to a bias in case selection. Data on the type of anesthesia and duration of surgery were not included in the database and could, therefore, not be evaluated. However, the duration of surgery may be derived from the type of surgery (primary vs. revision arthroplasty), which was equally distributed before and after the change in standard operative procedures. Despite the high number of patients, the number of events for some endpoints (e.g., periprosthetic joint infection) is low, for which no reliable conclusions can be made. Despite these limitations, the study demonstrates the effectiveness of a standard operative procedure change towards a more restrictive use of indwelling catheters in the prevention of UTI and, therefore, potentially periprosthetic joint infection.

## 4. Materials and Methods

All patients who underwent primary and revision arthroplasty at a German tertiary referral center for arthroplasty in 2021 and 2022 were selected and retrospectively included, with no exclusion criteria. Two groups were retrospectively analyzed: in 2021, routine use of indwelling catheters in perioperative treatment was the standard of care, whereas in 2022, the use was restricted, leading to a decrease in preoperative catheterization. In 2022, indications for indwelling catheters were limited to proximal femoral fractures with delayed surgical treatment due to the inability to ambulate and revise procedures based on the preferences of surgeons and anesthesiologists. The study was approved by our institutional review board and ethics committee (approval: 2022-3159-evBO).

Catheterization was performed under sterile conditions using Foley catheters. Patients who received indwelling catheters followed a standardized protocol that included daily evaluation of the possibility of catheter removal. Catheters were removed as soon as patients were able to ambulate and use the toilet or, at the latest, 48 h after surgery. All patients were monitored for urinary retention based on patient-endorsed symptoms and ultrasound bladder scans. Urinary retention was defined as the inability to empty the bladder volitionally within 6 h and a urine volume > 300 milliliters. In cases with a painful palpably distended bladder and a urine volume of >500 milliliters, postoperative urinary retention was defined independent of time after surgery. Patients with urinary retention were treated with in/out catheters once and, in case of persistent urinary retention, with a Foley catheter, and a urology consultation was obtained.

UTI was defined by the combination of clinical symptoms, leukocyturia, and positive urine culture. Positive urine cultures were defined as one or more bacteria in the urine at a growth of >100,000 colony-forming units per milliliter or >10,000 CFU per milliliter in the case of positive leukocyturia. Cultures with extended-spectrum beta-lactamase (ESBL) *Escherichia coli* were always defined as positive. In elderly patients, leukocyturia without clinical symptoms was not deemed sufficient for a UTI diagnosis. Patients receiving antimicrobial therapy for periprosthetic joint infection or UTI prior to surgery were excluded from the analysis for UTI. Antibiotic therapy was routinely started with intravenous cephalosporines and adapted to the antibiogram of the urine culture upon availability.

Statistical analysis was performed using SPSS version 29.0.0 (IBM, Armonk, NY, USA). Continuous variables were presented as mean (SD [standard deviation]; range), while nominal variables were expressed as frequencies (%). The groups were compared using the chi-squared test for categorical variables. Univariate logistic regression models were used to stratify variables associated with UTI, periprosthetic joint infection, and urinary retention. The influence of UTI and urinary retention on the length of hospital stay was analyzed using a multiple linear regression model. Multivariable logistic regression models were used to stratify risk factors associated with UTI and urinary retention. The independent variables were tested for multicollinearity and only included if the correlation was low (r < 0.7). Statistical significance of the models was reported with χ^2^ and *p*-value. Results of regression models were reported as odds ratio (OR), 95% confidence intervals (95% CI) and *p*-values. All tests were two-sided. *p* < 0.05 indicated statistical significance.

## 5. Conclusions

The use of indwelling catheters was associated with high rates of urinary tract infections (UTI), a previously published risk factor for periprosthetic joint infection. Restricting the use of indwelling catheters reduced UTI rates, with only a moderate increase in manageable cases of postoperative urinary retention.

Therefore, the use of indwelling catheters in primary arthroplasty should be avoided whenever possible to reduce the rates of UTI and potentially periprosthetic joint infection. In revision arthroplasty, especially acetabular cup revision, indwelling catheters reduce the rate of postoperative urinary retention. Nevertheless, due to the often devastating outcomes of periprosthetic joint infection in revision arthroplasty and reliable treatment options for urinary retention, indwelling catheters should only be used after careful consideration of the risks and benefits.

## Figures and Tables

**Table 1:** Patient demographic and surgical data.

	2021 (n = 752)	2022 (n = 769)	Total (n = 1521)
Mean age, years (SD; range)	70.6 (12.1; 27–100)	71.8 (11.0; 34–99)	71.2 (11.6; 27–100)
Sex, female/male, n (%)	403 (53.6)/349 (46.4)	437 (56.8)/332 (43.2)	840 (55.2)/681 (44.8)
Indwelling catheter, n (%)	466 (62.0)	292 (38.0)	758 (49.8)
Length of hospital stay, days (SD; range)	12.0 (8.3; 0–84)	11.7 (9.3; 0–116)	11.9 (8.8; 0–116)
Region			
Hip, n (%)	445 (59.2)	461 (59.9)	906 (59.6)
Knee, n (%)	307 (40.8)	308 (40.1)	615 (40.4)
Side			
Right, n (%)	370 (49.2)	394 (51.2)	764 (50.2)
Left, n (%)	382 (50.8)	375 (48.8)	757 (49.8)
Type of surgery			
Primary, n (%)	309 (41.1)	327 (42.5)	636 (41.8)
Revision, n (%)	332 (44.1)	314 (40.8)	646 (42.5)
Fracture, n (%)	111 (14.8)	128 (16.6)	239 (15.7)

**Table 2:** Multivariable logistic regression for urinary tract infection (UTI). Odds ratio (OR), 95% confidence intervals (95% CI) and *p*-values are reported for each variable. For the categorial variable type of surgery, primary arthroplasty was selected as the reference category.

Variable	OR	95% CI	*p*-Value
Age	1.03	0.99–1.06	0.170
Gender: female	0.64	0.30–1.38	0.253
Urinary catheterization	3.45	1.53–7.74	0.003 *
Region of surgery: hip	9.09	1.43–1.67	0.011 *
Type of surgery: revision vs. primary	0.72	0.31–1.67	0.441
Type of surgery: fracture vs. primary	0.39	0.15–1.04	0.059

* The numbers marked in bold indicate they are statistically significant (*p* < 0.05).

**Table 3:** Microbiological data of n = 34 patients with urinary tract infections (UTIs). Eight patients with postoperative UTI treated for periprosthetic joint infection were excluded from the analysis. Resistance patterns are included in Supplementary Table S1.

	UTI Cases (n = 34)
**Number of bacterial isolates per patient, n (%)**	
One bacterium	29 (85)
Two bacteria	5 (15)
Three or more bacteria	0
**Bacterial species, n**	
*Escherichia coli* (ESBL)	17 (5)
*Proteus mirabilis*	5
*Enterobacter cloacae*	3
*Klebsiella pneumoniae*	3
*Enterococcus faecalis*	2
*Pseudomonas aeruginosa*	2
*Citrobacter koseri*	1
*Serratia marcescens*	1

ESBL: Extended-spectrum beta-lactamases.

**Table 4:** Multivariable logistic regression for postoperative urinary retention. Odds ratio (OR), 95% confidence intervals (95% CI) and *p*-values are reported for each variable. For the categorial variable type of surgery, primary arthroplasty was selected as the reference category.

Variable	OR	95% CI	*p*-Value
Age	0.99	0.95–1.03	0.595
Gender: female	1.31	0.50–3.40	0.583
Urinary catheterization	0.02	0.003–0.17	<0.001 *
Region of surgery: hip	2.17	2.82–166.67	0.003 *
Type of surgery: revision vs. primary	16.02	4.47–57.44	<0.001 *
Type of surgery: fracture vs. primary	1.58	0.25–9.86	0.625

* The numbers marked in bold indicate they are statistically significant (*p* < 0.05).

**Table 5:** Indications and surgical procedures of all patients and patients with urinary tract infection (UTI; n = 42) and urinary retention (n = 21).

	Total	UTI	Urinary Retention
**Indication for surgery, n(%)**			
Coxarthrosis	328 (21.6)	13 (38.2)	2 (9.5)
Gonarthrosis	294 (19.3)	3 (8.8)	1 (4.8)
Femoral neck fracture	239 (15.7)	7 (20.6)	2 (9.5)
Periprosthetic joint infection	339 (22.3)	8 ^†^	2 (9.5)
Aseptic loosening	122 (8.0)	2 (5.9)	7 (33.3)
Arthrofibrosis/Ossification	13 (0.9)	0	0
Cut-out of proximal femur nail	14 (0.9)	1 (2.9)	0
Dislocation of total hip arthroplasty	33 (2.2)	1 (2.9)	4 (19.0)
Periprosthetic fracture	97 (6.4)	7 (20.6)	0
Polyethylene wear	16 (1.1)	0	3 (14.3)
Other	26 (1.7)	0	0
**Surgical procedure, n (%)**			
Total hip arthroplasty	483 (31.8)	17 (50.0)	3 (14.3)
Total knee arthroplasty	294 (19.3)	3 (8.8)	1 (4.8)
Hemiarthroplasty	98 (6.4)	4 (11.8)	1 (4.8)
Revision periprosthetic joint infection	339 (22.3)	8 ^†^	2 (9.5)
Revision hip arthroplasty: stem	62 (4.1)	5 (14.7)	0
Revision hip arthroplasty: cup	60 (3.9)	2 (5.9)	11 (52.4)
Revision hip arthroplasty: cup and stem	34 (2.2)	1 (2.9)	1 (4.8)
Osteosynthesis *	40 (2.6)	0	0
Exchange mobile components/ Arthrolysis	69 (4.5)	1 (2.9)	2 (9.5)
Revision knee arthroplasty	42 (2.8)	1 (2.9)	0

* Osteosynthesis for periprosthetic fractures includes implant revision (e.g., intraoperative evaluation of implant loosening and exchange of mobile parts). ^†^ Patients with periprosthetic joint infection were not included in the analysis for urinary tract infections.

## Data Availability

The data presented in this study are available upon request from the corresponding author due to ethical restrictions.

## Supplementary Materials

The following supporting information can be downloaded at: https://www.mdpi.com/article/10.3390/antibiotics14040368/s1, Table S1: Resistance patterns of the causative agents of urinary tract infection.
