# Supplementary material for: Indwelling Catheters Should Be Restricted in Primary and Revision Arthroplasty: A Retrospective Analysis After Changes to Hospital Standard Perioperative Treatment Protocol
Source: Antibiotics (Basel). 2025 Apr 2;14(4):368. doi: 10.3390/antibiotics14040368 (PMC12024275; doi:10.3390/antibiotics14040368)
Supplement: Supplementary file 1 [file antibiotics-14-00368-s001.zip › antibiotics-3426764-supplementary.pdf]

antibiotics

MDPI

Article

Indwelling Catheters Should Be Restricted in Primary and Revision Arthroplasty: A Retrospective Analysis After Changes to Hospital Standard Perioperative Treatment Protocol

Supplementary Table S1. Resistance patterns of the causative agents of urinary tract infection (UTI). Reported data shown are taken from the antibiograms according to the Clinical & Laboratory Standards Institute (CSLI). In cases of intermediate resistance, results are reported as number of resistant pathogens/number of pathogens with intermediate resistance.

Bacterial species (n)	Number specimen with antimicrobial resistance																						
	AMP	SAM	AMC	PIP	TZP	CXM	CTX	CRO	CAZ	CPM	ETP	IPM	GEN	AMK	TET	SXT	VAN	FO	CIP	LVX	MFX	NIT	CMS	NTX
Escherichia coli (non-EBSL; 12)	9	2/4	2/4	7	0	0	0	0	0	0	0	0	0	0	2	1	-	1	2	2	5	1	2/1	0
Escherichia coli ESBL (5)	5	5	5	5	1	5	5	5	5	5	0	0	0	0	3	2	-	2	5	5	5	0	0/3	0
Proteus mirabilis (5)	3	0/1	0/1	1/1	0	0	0	0	0	0	0	1/2	0	1	5	2	-	2	0	0	0	5	5	1
Enterobacter cloacae (3)	3	2	2	2	1	2	1	1	1	0	0/1	0	0	0	0	0	-	0	0	0	0	0/1	0	0
Klebsiella pneumoniae (3)	3	1	1	1	0	1	0	0	0	0	0	0	0	0	0	0	-	0	0	0	0	0/1	0	0
Enterococcus faecalis (2)	0	0	0	-	0	-	-	-	-	-	-	0	-	-	0	-	0	-	0	0	0	0	-	-
Pseudomonas aeruginosa (2)	-	-	-	1	0	-	-	-	0	0	-	0	0	0	-	-	-	2	0	0	-	-	0	-
Citrobacter koseri (1)	1	1	1	1	0	1	0	0	0	0	0	0	0	0	0	0	-	0	0	0	0	0	0	0
Serratia marcescens (1)	1	1	1	1	0	1	1	1	0	0	0	0	0	1	1	0	-	0	0	0	0	1	1	0

AMP: Ampicillin; SAM: Ampicillin-Sulbactam; AMC: Amoxicillin/Clavulanic acid; PIP: Piperacillin; TZP: Piperacillin-Tazobactam; CXM: Cefuroxime; CTX: Cefotaxime; CRO: Ceftriaxone; CAZ: Ceftazidime; CPM: Cefepime; ETP: Ertapenem; IPM: Imipenem; GEN: Gentamicin; AMK: Amikacin; TET: Tetracycline; SXT: Trimethoprim/Sulfamethoxazole; FO: Fosfomycin; CIP: Ciprofloxacin; LVX: Levofloxacin; MFX: Moxifloxacin; NIT: Nitrofurantoin; CMS: Colistin; NTX: Nitroxoline

 https://doi.org/10.3390/antibiotics14040368
